# Evaluating Degenerative Lumbar Disease with Markerless 3D Motion Capture: Reliability and Validity in Sit-to-Stand Test

**DOI:** 10.3390/s25103122

**Published:** 2025-05-15

**Authors:** Yi-Ting Huang, Szu-Hua Chen, Chao-Ying Chen, Shiu-Min Wang, Pei-Yuan Wu, Dar-Ming Lai, Wei-Li Hsu

**Affiliations:** 1 School and Graduate Institute of Physical Therapy, College of Medicine, National Taiwan University, No. 17, Xuzhou Rd., Zhongzheng Dist., Taipei City 100, Taiwan; r11428004@ntu.edu.tw (Y.-T.H.); d09428002@ntu.edu.tw (S.-M.W.); 2 Department of Physical Therapy, Ithaca College, Ithaca, NY 14850, USA; schen3@ithaca.edu; 3 Physical Therapy Centre, National Taiwan University Hospital, No. 1, Changde St., Zhongzheng Dist., Taipei City 100, Taiwan; ccypt@ntu.edu.tw; 4 Department of Electrical Engineering, National Taiwan University, Taipei City 106, Taiwan; peiyuanwu@ntu.edu.tw; 5 Division of Neurosurgery, Department of Surgery, National Taiwan University Hospital, Taipei City 100, Taiwan; dmlai@ntu.edu.tw

**Keywords:** degenerative lumbar disease, sit-to-stand test, markerless motion capture system

## Abstract

Background: Degenerative lumbar disease (DLD) affects older adults, causing lumbar degeneration and lower extremity dysfunction. The five-times sit-to-stand test (5xSTS) reveals kinematic changes associated with DLD. While marker-based motion capture systems detect these changes, their complexity limits clinical use. Markerless motion capture offers a portable alternative, yet its functional assessment applications in DLD remain underexplored. Thus, the aim of this study is to evaluate the reliability and validity of markerless motion capture for assessing functional tests in DLD patients. Methods: This study included 11 healthy individuals (mean age: 27.28 ± 6.92 years) and 10 with DLD (mean age: 70.00 ± 8.08 years). Participants performed the 5xSTS while being recorded by marker-based (VICON) and markerless (MediaPipe) systems using two synchronized cameras. Test–retest reliability was assessed over one week via the intraclass correlation coefficient (ICC). Concurrent validity and agreement between VICON and MediaPipe were evaluated via Pearson/Spearman correlation coefficients, systematic bias, and the root mean square error (RMSE). Movement time, joint excursions, and angular velocities were also analyzed and compared across two groups. Results: Both systems showed high test–retest reliability (ICC: 0.81–0.99) and strong correlations (r: 0.75–0.99). The highest RMSE was observed at the ankle in the anterior–posterior (A–P) direction in the DLD group (54.55 mm) and at the hip A–P axis in the control group (51.20 mm). The lowest RMSE was found at the knee medial–lateral (M–L) axis in the DLD group (7.88 mm) and at the ankle M–L axis in the control group (8.54 mm). Bias values ranged from 0.30 mm (hip vertical in control group) to +53.47 mm (ankle A–P in DLD group), with underestimation more common at the hip and overestimation at the ankle. The control group demonstrated a faster 5xSTS completion time (5.89 ± 0.69 s vs. 8.13 ± 1.96 s, *p* < 0.05), greater hip joint excursions during sit-to-stand (65.07 ± 25.94° vs. 38.13 ± 9.84°, *p* < 0.05) and stand-to-sit (62.56 ± 24.74° vs. 27.85 ± 11.45°, *p* < 0.05) tests, and higher angular velocities compared to the DLD group. Conclusion: MediaPipe markerless motion capture can quantify 3D kinematic changes in DLD patients during functional performance. It enables a clinical evaluation with minimal setup, offers potential for remote assessment, and accurately detects sagittal plane movement. The two-camera system provides 3D kinematic data comparable to multi-camera systems, suitable for home rehabilitation and assessment.

## 1. Introduction

Degenerative lumbar disease (DLD) involves pathoanatomical abnormalities in the lumbar spine, including disc degeneration, vertebral osteophytes, facet joint arthrosis, and ligamentous hypertrophy [1,2]. These abnormalities lead to symptoms such as pain, muscle weakness, neurogenic claudication, and impaired balance [3,4]. The pathologies in individuals with DLD significantly impact functional performance and increase disability [5,6].

In clinical practice, clinicians commonly use the five-times sit-to-stand (5xSTS) test to measure functional performance in individuals with DLD [7]. A systematic review identified it as a core clinical measure, with nine studies involving 955 participants using the 5xSTS to evaluate physical performance in this population [8]. The 5xSTS involves instructing participants to stand up and sit down five times as quickly as possible without using their hands. Success on this test depends on sufficient joint torques, lower extremity strength, sensorimotor coordination, balance, and cognitive abilities [9]. Sit-to-stand transitions involve significant demands on the hip and trunk, making them sensitive to the kinematic changes associated with DLD, such as decreased hip range of motion (e.g., 51.0° vs. 77.25° in healthy controls) and slower hip angular velocity (46°/s vs. 69°/s), along with reduced trunk acceleration [10]. Additionally, in individuals with DLD, limited lumbar and hip mobility results in greater movement variability [11] and longer movement time [5,10,12,13]. These findings highlight the need for precise motion analysis tools that can detect such subtle kinematic changes.

The 5xSTS is commonly timed with a stopwatch [14], providing temporal data. However, this method overlooks the detailed kinematic changes essential for a comprehensive evaluation. The kinematic changes are typically measured using marker-based motion capture systems in the laboratory, a method impractical for clinical use due to its complexity. On the other hand, the markerless motion capture systems offer several advantages over traditional marker-based systems. First, it reduces the preparation time and optimizes the comfort for the participants as it eliminates the need for reflective markers on the bony landmarks [15]. Secondly, it significantly reduces data processing time for gap filling and motion tracking through advanced algorithms and recognition technology [15]. The markerless systems also provide clinical feasibility through their portability, allowing for assessments outside of specialized laboratory environments. Overall, markerless motion capture is simpler, faster, and more user-friendly, enhancing efficiency and convenience across various fields such as sports, rehabilitation, and academic research [16,17].

Markerless human pose estimation usually utilizes an RGB-D camera to acquire 2D motions, such as Microsoft Kinect. However, using a single camera to capture the depth through infrared light typically results in poor depth accuracy. Recently, the advancement of human pose estimation models have relied on deep learning to interpret the depth from RGB images, including but not limited to OpenPose (2016), AlphaPose (2017), and MediaPipe (2019) [18,19]. OpenPose has high operational requirements for device performance, leading to prolonged processing time and high latency [15]. AlphaPose is not feasible for mobile use and is limited to high-performance Windows or Linux systems [20,21]. MediaPipe, utilizing BlazePose, performs efficiently across platforms with minimal processing times, low latency, and high frame rates, making it the optimal choice for platform compatibility and reduced operational requirements and processing times [22,23]. To summary, MediaPipe offers several practical advantages: (1) it requires no additional hardware beyond video cameras, enabling easy and low-cost implementation; (2) it supports cross-platform operation, making it well-suited for portable and remote applications; and (3) it offers short processing times with minimal system performance requirements. These considerations make MediaPipe a more accessible option for clinical applications.

Functional movements are inherently three-dimensional. The single-camera models, limited to the sagittal plane, provide accurate 2D information but lack 3D accuracy. Therefore, using a dual-camera MediaPipe model to obtain 2D pose estimations with the application of triangulation is essential to reconstruct 3D information accurately. Therefore, in this study, a dual-camera MediaPipe model was used to estimate 2D body landmarks from synchronized video streams. To reconstruct 3D poses, triangulation was applied by matching corresponding landmarks from the two views. The 3D coordinates were computed based on the geometric relationship between the cameras, enabling the accurate representation of 3D human movement.

Although markerless motion capture systems like MediaPipe can obtain both temporal and 3D spatial data and objectively quantify the kinematic changes during functional performance, only a few studies have used such systems in patient populations. Thus, this study aimed to establish the reliability and validity of using a markerless motion capture system to examine the 3D lower extremity kinematics during the five-times sit-to-stand test in individuals with DLD and compare the data between individuals with DLD and healthy controls to validate its application. We hypothesized that the MediaPipe motion capture system would demonstrate good to excellent test–retest reliability in capturing lower extremity kinematics during the 5xSTS. Furthermore, we expected that individuals with DLD would exhibit significantly different kinematic patterns, such as reduced joint excursion or altered movement strategies, thereby supporting the discriminative validity of the system.

## 2. Materials and Methods

### 2.1. Study Design

This cross-sectional study evaluated the reliability and validity of the markerless motion capture system in young adults and its application in individuals with DLD. It was approved by the Research Ethics Committee of University Hospital (202201047RINC) and registered on ClinicalTrials.gov (NCT05425680).

### 2.2. Participants Recruitment

Participants included healthy adults and individuals with DLD. Inclusion criteria were as follows: (1) aged 20–80 years and (2) able to sit and stand independently. Exclusion criteria were as follows: (1) history of lower extremity surgery, (2) neurological disorders, (3) diabetes or vestibular disease, and (4) ankylosing spondylitis or rheumatoid arthritis. Participants gave informed consent upon enrollment.

### 2.3. Data Collection and Analysis

Demographic and anthropometric data were collected, including age, sex, height, weight, leg length, knee width, and ankle width. Leg length was measured with a tape measure, while the joint widths were assessed with a caliper.

### 2.4. Experimental Setup

Participants underwent two visits in a laboratory equipped with both marker-based and markerless motion capture systems. Two visits were one week apart to establish test–retest reliability. Participants received a detailed explanation of the experimental procedures and provided informed consent prior to testing. A trained graduate student from the Department of Physical Therapy, who was experienced in administering the 5xSTS and operating both motion capture systems, conducted all measurements to ensure consistency across participant data. Participants were instructed to wear form-fitting clothing and perform the 5xSTS. During the 5xSTS, participants were seated on a standard-height chair (approximately 43 cm) without armrests, with feet flat on the floor and hip-width apart. They were instructed as follows: “Stand up and sit down as quickly as possible, five times. Place your hands on your thighs and avoid utilizing them throughout the entire procedure. Rest your back against the backrest of the chair at the end of each movement”. Timing began on the command “Go” and stopped when the participant’s back touched the backrest after the fifth repetition. To minimize learning effects and improve reliability, each participant completed two practice trials of the 5xSTS prior to the recorded trial. These practice trials served as a warm-up protocol and ensured participants fully understood the testing instructions and procedure. The 5xSTS has been shown to be a reliable and valid measure of lower extremity function [14].

**Figure 1:**
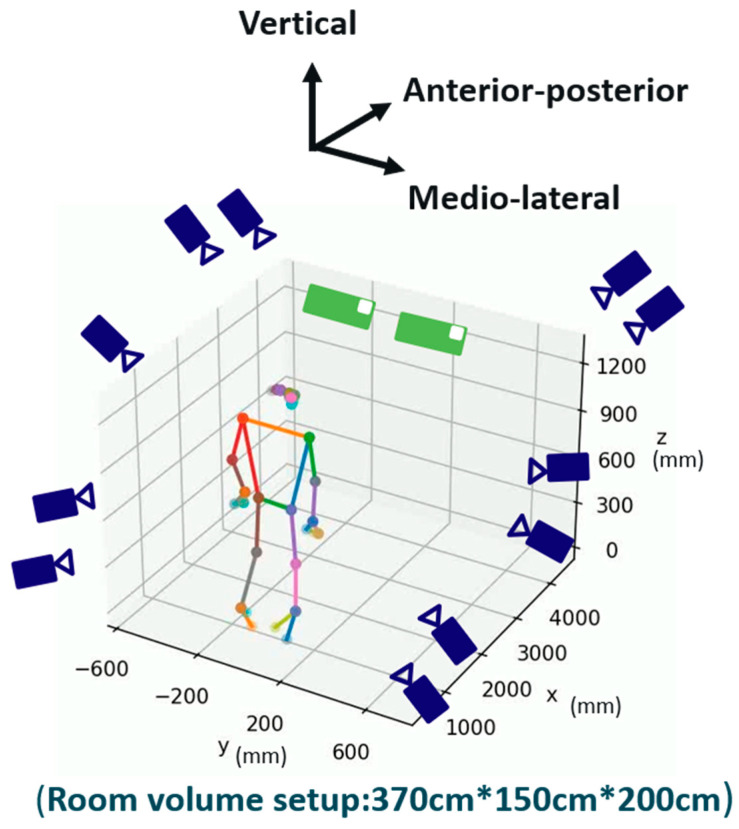
Experimental setup consisting of an 11-camera VICON motion capture system and two iPhone 14 devices.

Movements were recorded using an 11-camera VICON system (ver. 2.5, Oxford Metrics Ltd., Oxford, UK) and two iPhone 14 mobile phones (iOS ver. 16.0.1, Apple Inc., Cupertino, CA, USA). The VICON system (sampling rate: 120 Hz) used 45 reflective markers to capture joint kinematics, while the MediaPipe system (ver. 0.10.3, Google, CA, USA) used two iPhone 14 cameras to estimate 3D poses from 2D images. The cameras captured synchronized videos, which were processed to reconstruct 3D poses using triangulation. The iPhone cameras and the VICON system were not hardware-synchronized during recording. Instead, data alignment was performed manually by calculating the Mean Per Joint Position Error (MPJPE) across multiple potential start times. The start time that yielded the lowest MPJPE was selected as the reference point for alignment between the two systems. Video data from the two iPhone 14 devices were captured simultaneously using the iVCam app (ver. 7.2, Shanghai Yitu Information Technology Co., Ltd., Shanghai, China) and synchronized using Open Broadcaster Software (OBS ver. 28.1.2). The cameras were spaced 40 cm apart and aligned in parallel, centered on the subject to maintain a consistent frontal view and to simplify the setup. Both devices were mounted on tripods at a height of approximately 90 cm from the ground to ensure stability and consistency across participant data. Although a parallel placement provides less angular disparity compared to convergent setups, triangulation was still achieved effectively due to the fixed baseline distance and frontal positioning. This configuration balances ease of use with the ability to capture accurate 3D joint positions in the sagittal plane. The system’s simplicity and portability allow for rapid deployment in clinical environments and open the possibility for home-based assessments. While a dual-camera setup may require minimal technical assistance for an initial configuration at home, it still provides a feasible and scalable approach for telemedicine applications in rehabilitation. The experimental setup is shown in Figure 1.

### 2.5. Joint Kinematic Data

Joint position data from MediaPipe and VICON systems were collected during the 5xSTS. Movement time was recorded, and the sit-to-stand movement was segmented into three events: start (5% of initial displacement), maximum (velocity approaches zero), and end (5% of displacement during the stand-to-sit phase) [24,25]. The reliability and validity of the MediaPipe model via the markerless motion system during these events were evaluated. MediaPipe’s BlazePose model tracks 33 anatomical landmarks across the body. For this study, we focused on the hip and knee joints. Joint angles were calculated using the 3D coordinates of relevant landmarks, obtained through triangulation from the synchronized dual-camera video. Angles were estimated by using a geometric method based on the law of cosines, which calculates the angle formed by three anatomical landmarks. Joint excursions and angular velocities of the hip and knee joints were then computed during both the sit-to-stand and stand-to-sit phases using a customized MATLAB (R2024b) program.

### 2.6. Statistical Analysis

Descriptive data of the participants were presented as means and standard deviations for continuous data. The Shapiro–Wilk test was employed to determine the normality of the difference between the MediaPipe and VICON data.

To evaluate test–retest reliability, a two-way mixed-effects intraclass correlation coefficient (ICC) model with absolute agreement for single measures was used. The levels of reliability were categorized as slight (0.00–0.20), fair (0.21 to 0.40), moderate (0.41 to 0.60), substantial (0.61 to 0.80), and almost perfect (0.81 to 1.00) [26].

To assess the validity of MediaPipe in estimating joint positions compared to the VICON system, several complementary metrics were used, including the root mean square error (RMSE) to quantify the magnitude of the positional error and the mean bias to determine average differences between systems. A correlation analysis was also performed. Pearson’s correlation coefficient with 95% confidence intervals was reported for normally distributed data, and Spearman’s rank correlation coefficient was reported for non-normally distributed data. The correlation strength, represented as ‘r’, was categorized as negligible (0.00 to 0.30), low (0.30 to 0.50), moderate (0.50 to 0.70), high (0.70 to 0.90), and very high (0.90 to 1.00) [26].

In a clinical application, this study further compared the kinematic data of the lower limb joint angle and angular velocity between the individuals with DLD and the healthy control using MediaPipe. Parametric methods (Independent Samples *t*-test) and non-parametric methods (Mann–Whitney U test and Chi-square test) were employed for this part of the analyses, depending on the data distribution. All statistical significance was set at a *p*-value of less than 0.05. The PASW Statistics 25 for Windows software (SPSS 26, Chicago, IL, USA) was used for the statistical analyses.

Since the pilot data indicated that there were no significant differences in kinematics data between the left and right lower limb joints, the following results are presented for the right lower limb joints only.

## 3. Results

### 3.1. Demographic and Anthropometry Data

The demographic and anthropometry data are shown in Table 1. A total of 21 participants were recruited. Participants were divided into a healthy control group (*n* = 11) and a DLD group (*n* = 10). The DLD group was significantly older, shorter, and presented with a shorter leg length.

### 3.2. Test–Retest Reliability

The results of test–retest reliability are presented in Table 2. The reliability of joint positions was remarkably high. The hip joint exhibited the highest consistency, with ICC values ranging from 0.94 to 0.99, followed by the knee joint, which also demonstrated reliability, ranging from 0.86 to 0.99. The ankle joint position showed slightly lower consistency than the hip and knee joints, with ICC values ranging from 0.81 to 0.98 and a larger range of the 95% confidence interval. Thus, the consistency of joint positions followed the following order: hip joint > knee joint > ankle joint. Moreover, on the anterior–posterior (A–P) axis of the hip, knee, and ankle joints, the maximum event of the sit-to-stand event exhibited greater consistency compared to the start and end event (hip: 0.98 > 0.97; knee: 0.97 > 0.87; ankle: 0.91 > 0.81). On the medio–lateral (M–L) axis of the hip, knee, and ankle joints, the start event of the sit-to-stand movement exhibited greater consistency than the maximum and end event (hip: 0.99 > 0.94; knee: 0.99 ≥ 0.98; ankle: 0.98 > 0.81). However, in the vertical axis of the hip joint, the end event of the sit-to-stand movement exhibited better consistency than the start and maximum points (hip: 0.97 > 0.95), while in the knee and ankle joints, the maximum event demonstrated greater consistency than the start and end events (knee: 0.99 > 0.93; ankle: 0.88 > 0.83).

### 3.3. Concurrent Validity

The validity data comparing the MediaPipe system to the VICON system is presented in Table 3 and Table 4. Both the control group and the DLD group exhibited high to very high correlations between two motion systems, with all *p*-values below 0.01. In the control group (Table 3), the correlations between the two systems at the hip joint ranged from 0.89 to 0.99, while the correlations ranged from 0.96 to 0.99 and 0.85 to 0.97 for the knee joint and ankle joint, respectively. In the DLD group (Table 4), the correlations between the two systems at the hip joint ranged from 0.86 to 0.98, the values ranged from 0.84 to 0.99 for the knee, and from 0.87 to 0.99 for the ankle joint.

Overall, the control group (*r* = 0.85 to 0.99) exhibited better Pearson correlation coefficient values than the DLD group (*r* = 0.84 to 0.99), particularly in the hip and knee joints. In the control group, the hip and knee joints showed better performance on the A–P axis compared to the M–L and vertical axes (hip: 0.99 > 0.89; knee: 0.99 > 0.98), while in the ankle joint, the vertical axis performed better (ankle: 0.97 > 0.85). For the DLD group, the vertical axis performed better performance than the other two axes across all joints (hip: 0.99 > 0.86; knee: 0.99 > 0.85; ankle: 0.99 > 0.87).

### 3.4. Agreement Analysis

Across both groups, RMSE values were consistently higher along the anterior–posterior axis compared to the vertical and medial–lateral axes. Within the A–P and M–L directions, the hip joint exhibited the highest RMSE values overall, particularly in the control group. In contrast, within the vertical axis, the knee showed the greatest RMSE during the start and end phases, while the ankle exhibited the highest RMSE during the maximum phase. The largest RMSE in the control group occurred at the hip joint in the A–P direction (51.20 mm), whereas in the DLD group, the ankle A–P axis had the highest RMSE (54.55 mm). The lowest RMSE values were found at the ankle M–L axis in the control group (8.54 mm) and at the knee M–L axis in the DLD group (7.88 mm). These findings suggest that MediaPipe’s positional accuracy is highest in the M–L direction and at distal joints, while tracking variability increases in the A–P direction, particularly at the hip and ankle during dynamic movement phases.

Bias values varied across joints, axes, and movement phases. In the control group, the largest bias was observed at the hip joint in the anterior–posterior (A–P) axis (–49.89 mm), indicating that MediaPipe substantially underestimated the hip position in this direction. The smallest bias in the healthy group was found at the hip vertical axis (0.30 mm). In the DLD group, the largest bias occurred at the ankle joint in the A–P axis (+53.47 mm), suggesting significant overestimation, while the smallest bias was observed at the hip vertical axis (0.42 mm). Overall, underestimation was more common at the hip joint across both groups, while overestimation was more frequently seen at the ankle joint.

### 3.5. Joint Kinematic Data

**Figure 2:**
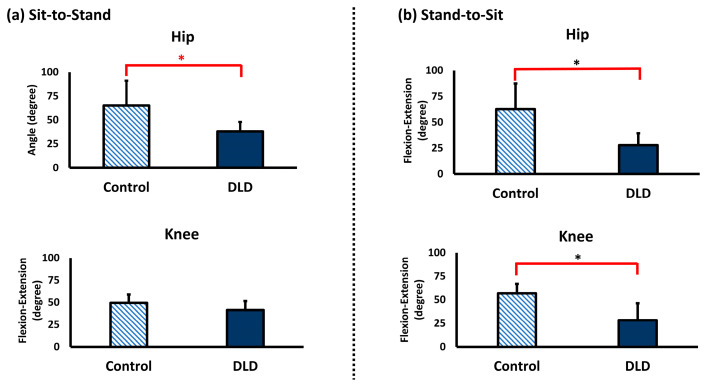
Joint excursion during five-times sit-to-stand movement of the control and DLD (degenerative lumbar disease) groups. * indicates significant differences between groups.

**Figure 3:**
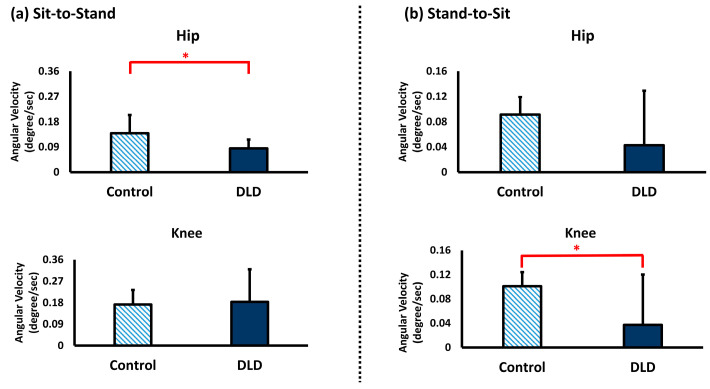
Angular velocity during five-times sit-to-stand movement of the control and DLD (degenerative lumbar disease) group. * indicates significant differences between groups.

The results of joint excursions and angular velocity during the 5xSTS of the control group and DLD group are shown in Figure 2 and Figure 3.

The movement time for the 5xSTS in the control group (mean = 5.89 ± 0.69 s) was significantly shorter compared with that of the DLD group (mean = 8.13 ± 1.96 s) (*p* < 0.05).

The joint excursion of the hip joint from sitting to standing was significantly greater in the control group (mean = 65.07 ± 25.94 degrees) compared to the DLD group (mean = 38.13 ± 9.84 degrees) (*p* < 0.05). However, there was no significant difference in the knee joint. Similarly, during the stand-to-sit movement, the joint excursion of the hip joint was significantly greater in the control group (mean = 62.56 ± 24.74 degrees) compared to the DLD group (mean = 27.85 ± 11.45 degrees) (*p* < 0.05). Additionally, the joint excursion of the knee joint in the control group (mean = 56.76 ± 10.25 degrees) was significantly greater than in the DLD group (mean = 28.22 ± 18.05 degrees) (*p* < 0.05).

The angular velocity of the hip joint during the sit-to-stand movement in the control group (mean = 0.14 ± 0.07 degrees/s) was significantly greater than that in the DLD group (mean = 0.09 ± 0.03 degrees/s) (*p* < 0.05), but there was no significant difference in the knee joint. During the stand-to-sit movement, there was no significant difference in the angular velocity of the hip joint in the control and DLD groups. However, the angular velocity of the knee joint in the control group (mean = 0.10 ± 0.02 degrees/s) was significantly greater than in the DLD group (mean = 0.03 ± 0.08 degrees/s) (*p* < 0.05).

## 4. Discussion

This study aimed to investigate the reliability and validity of the MediaPipe system for the 5xSTS. We found that MediaPipe achieved good reliability and validity performance in healthy adults and DLD patients. These results focused on the potential of MediaPipe as an effective tool for evaluating kinematic data in individuals with DLD, such as movement time, joint excursion, and angular velocity data.

### 4.1. Test–Retest Reliability

In this study, besides the ankle joint, most joints in the lower extremities showed high consistency between the test and retest. The inferior consistency observed in the ankle joint might be attributed to a relatively small range of ankle motion during the 5xSTS. Similar findings were observed in a study that incorporated a Kinect system for the motion analysis [11]. This study noted a diminished accuracy in the ankle joint and attributed the result to the challenge of detecting and differentiating the ground and ankle movements due to the ankle’s relatively smaller range of motion [27]. Specifically, the signal-to-noise ratios (SNRs) might be low, to the extent similar to the amplitude of the movement [11]. On the other hand, the high reliability on both the anterior–posterior and vertical axes might be due to the fact that the large amplitude of the joint motion occurred during the 5xSTS along these two axes, resulting in a high SNR.

### 4.2. Concurrent Validity and Agreement

Our study found a significant correlation between the MediaPipe and VICON systems in both groups, confirming the validity of MediaPipe for evaluating lower limb kinematics. However, the control group showed higher validity performance than the DLD group, especially in the hip and knee joints. This indicated that MediaPipe may work better with consistent and less variable movement patterns, which are typical of healthy participants. In contrast, the greater variability and complexity of movements in the DLD group may present relative challenges in the validity.

In the control group, the hip and knee joints showed better validity on the anterior–posterior and vertical axes than the medio–lateral axis. This could be attributed to the nature of the 5xSTS, where movements mainly occur in the sagittal plane. In the DLD group, the lower extremity joints showed better validity on the vertical axis than the other two axes, indicating that MediaPipe captured the vertical movements more consistently with the traditional marker-based motion system. It is plausible that the DLD group presented with high variability in movements along with the anterior–posterior and medio–lateral axes due to clinical symptoms [28]. Specifically, the DLD group may experience back pain during the sit-to-stand process, thus limiting trunk forward and backward movement [29]. A previous study also indicated that the DLD group had poor postural medial–lateral stability, as evidenced by an increased sway of the center of pressure in the medio–lateral direction [30].

The validity assessment of our study along the anterior–posterior axis was consistent with previous motion analysis studies using Kinect and Vicon [6,11]. Furthermore, our study demonstrated superior performance on both the medio–lateral and vertical axes compared to the aforementioned study. This study proved that MediaPipe accurately detects movement in the sagittal plane, which is a limitation of other commercial motion analysis systems.

The RMSE and bias patterns observed in this study demonstrate both the strengths and limitations of MediaPipe for markerless motion tracking during functional movements like those in the 5xSTS. Errors were generally higher in the anterior–posterior axis, especially at the hip and ankle, reflecting the common challenge of depth estimation in 2D-based pose estimation models. Conversely, the medial–lateral axis and distal joints, such as the knee and ankle, showed lower RMSE values, indicating greater tracking consistency. When compared to previous work using OpenPose with a five-camera setup during walking [31], where the RMSE ranged from 6.41 mm (knee M–L) to 28.6 mm (ankle A–P), the RMSE values reported here were generally larger, likely due to differences in the number of cameras and movement complexity. Interestingly, the largest and smallest RMSE values reported in that prior study match those observed in our DLD group, suggesting that while the overall error may be higher in our setup, the extreme bounds of error are comparable across systems when assessing joint-specific performance. The bias analysis revealed that MediaPipe tended to underestimate positions at the hip and overestimate positions at the ankle, with variability depending on the movement phase and joint. These findings highlight the importance of interpreting markerless motion capture data in a joint- and direction-specific manner, particularly when used in clinical populations or for longitudinal tracking.

### 4.3. Clinical Application

Our study provides clinical evidence that the MediaPipe system can identify pathological movements, such as movement time, joint excursion, and angular velocity in patient populations. This current study showed the DLD group spent more time during the 5xSTS than the control group, which might indicate movement difficulties, potentially from muscle weakness, balance issues, or pain based on the study of Claeys et al. [32].

This current study also showed that the DLD group had decreased joint excursion. This is similar to our previous study, which found that aging people might use a stiff joint strategy for movement control, resulting in a limited range of motion in the DLD group [33,34]. As for the angular velocities, the DLD group had decreased angular velocities, possibly as a form of cautious protection or pain avoidance [35].

Taken together, the current study provides a simplified solution for motion analysis in patient populations. Several studies have used up to eight cameras to detect kinematic motion [36,37,38], but the complexity of these setups poses challenges for tele-rehabilitation and routine clinical use. The MediaPipe two-camera system provides three-dimensional kinematic data; along with its portability, affordability, and minimal setup, it allows clinicians to collect meaningful movement data without the need for specialized lab environments or expensive equipment. This approach has significant clinical implications, particularly in home-based or community-based settings, where access to motion analysis technology is often limited. By enabling the detection of subtle motor deficits and tracking progress over time, markerless systems have the potential to support early identification, individualized intervention planning, and remote monitoring in patient populations.

### 4.4. Study Limitation

This study has several limitations that should be considered when interpreting the findings. First, the markerless nature of the MediaPipe system introduces potential inaccuracies, particularly in capturing movements outside the sagittal plane. Although MediaPipe is capable of estimating three-dimensional joint positions, its accuracy diminishes in the frontal and transverse planes due to challenges in depth estimation and occlusion, leading to limb mislabeling or joint switching. Given that the sit-to-stand task primarily involves sagittal plane motion, our analysis focused solely on sagittal plane kinematics. While appropriate for the task, this approach limits insight into compensatory movements that may occur in other planes. Future studies should explore MediaPipe’s performance in more complex, multi-planar movements.

Second, although we implemented a standardized dual-camera setup and instructed participants to wear form-fitting clothing to minimize occlusion, we did not systematically evaluate how variations in camera angle, clothing, or environmental conditions such as lighting may impact tracking accuracy. These factors are known to affect vision-based systems, particularly in less controlled settings. Future studies should test the robustness of MediaPipe under variable real-world conditions and explore mitigation strategies for these sources of error.

Third, while synchronized cameras were used, we did not perform a detailed analysis of potential frame rate discrepancies or synchronization errors. Even small temporal misalignments between camera feeds can lead to spatial inaccuracies in 3D joint reconstruction. Future work should assess synchronization accuracy, evaluate the impact of temporal variability on kinematic outputs, and consider implementing hardware-based synchronization or post-processing correction methods to minimize temporal errors.

Fourth, the absence of spinal or pelvic landmarks in the MediaPipe BlazePose model limited the precision of hip joint angle estimation, especially during movements involving substantial trunk or pelvic motion. This anatomical gap may affect the accuracy of joint angle calculations. Future studies should consider integrating additional sensors or algorithmic strategies to estimate trunk and pelvic motion more accurately.

Fifth, the study included a relatively small sample size and a significant age difference between the DLD group (mean age ~70) and the healthy control group (mean age ~27). Although the sample size was determined through a priori power analysis, the demographic mismatch may introduce confounding effects in group comparisons and limits generalizability. Future research should aim to recruit larger, age-matched cohorts that better represent target clinical populations.

Sixth, data collection and analysis were performed by the same researcher, who was aware of participants’ group assignments. Although joint kinematics were generated through automated pipelines, the absence of blinding during data processing may have introduced unintentional bias, particularly in event segmentation or data quality decisions. Future studies should incorporate independent or blinded analysts to enhance objectivity and reduce potential bias.

Seventh, this study was limited to offline data processing and did not assess MediaPipe’s suitability for real-time applications. While this was sufficient for the current study’s objectives, the lack of real-time capability limits its use in clinical or performance settings where immediate feedback is essential. Future work should explore real-time processing pipelines, including algorithm optimization and hardware requirements, to expand the system’s applicability in time-sensitive environments.

Finally, although this study includes comprehensive validity and accuracy metrics, the variability observed across joints suggests that MediaPipe’s performance is task-, axis-, and joint-specific. Notably, concurrent validity showed strong correlations between MediaPipe and the marker-based system (*r* = 0.75–0.99), supporting the system’s potential for functional kinematic assessment. However, these findings should still be interpreted as preliminary. High correlation does not necessarily equate to clinical equivalence, particularly in the presence of systematic error. Additional validation across diverse movement tasks, populations, and environments is needed before MediaPipe can be considered ready for widespread clinical application.

## 5. Conclusions

This study provides preliminary evidence supporting the feasibility of using a dual-camera MediaPipe-based markerless motion capture system to assess lower extremity kinematics during the five-times sit-to-stand task in individuals with degenerative lumbar disease (DLD). To our knowledge, this is the first study to apply a markerless motion capture system for clinical evaluation in this population. This system demonstrated high reliability and strong concurrent validity with a marker-based system. Markerless systems offer practical advantages, including reduced setup time, minimal equipment requirements, and user-friendly application, making them promising tools for use in clinical and telemedicine settings. However, variability in accuracy across joints and movement axes, along with observed systematic bias, suggests that MediaPipe’s performance remains task- and joint-specific. These findings support the potential of markerless motion capture in clinical evaluation, but further validation across a broader range of functional tasks, patient populations, and real-world environments is needed to establish its clinical readiness.

## Figures and Tables

**Table 1:** Demographic and anthropometry data of the participants.

Characteristics		Control Group (*n* = 11)	DLD Group (*n* = 10)	*p*-Value ^a,b^
Age (year)		27.28 ± 6.92	70.00 ± 8.08	*p* < 0.01 ^a^
Height (cm)		169.73 ± 7.88	158.50 ± 8.34	*p* < 0.01 ^a^
Weight (kg)		66.18 ± 12.81	63.00 ± 17.63	*p* = 0.65 ^a^
Sex (men/women)		8/3	3/7	*p* = 0.05 ^b^
Leg length (cm)	Right	86.79 ± 3.89	82.80 ± 4.41	*p* = 0.04 ^a^
	Left	86.58 ± 4.46	82.10 ± 4.98	*p* = 0.04 ^a^
Knee width (cm)	Right	10.85 ± 0.72	11.18 ± 0.99	*p* = 0.41 ^a^
	Left	10.85 ± 0.71	11.22 ± 0.99	*p* = 0.35 ^a^
Ankle width (cm)	Right	7.03 ± 0.56	6.51 ± 0.51	*p* = 0.57 ^a^
	Left	6.96 ± 0.54	6.48 ± 0.55	*p* = 0.58 ^a^

Values are mean ± SD or number. ^a^ Independent Samples *t*-test; ^b^ Chi-square test.

**Table 2:** Test–retest reliability of MediaPipe for joint positions.

Joint	Axis	Event	ICC	95% CI
Hip	A–P	Start	0.97	0.89~0.99
		Maximum	0.98	0.92~0.99
		End	0.97	0.90~0.99
	M–L	Start	0.99	0.97~0.99
		Maximum	0.94	0.76~0.98
		End	0.96	0.84~0.99
	Vertical	Start	0.96	0.85~0.99
		Maximum	0.95	0.87~0.99
		End	0.97	0.87~0.99
Knee	A–P	Start	0.86	0.47~0.96
		Maximum	0.97	0.90~0.99
		End	0.87	0.50~0.96
	M–L	Start	0.99	0.98~0.99
		Maximum	0.99	0.98~0.99
		End	0.98	0.98~0.99
	Vertical	Start	0.93	0.74~0.98
		Maximum	0.99	0.99~1.00
		End	0.96	0.88~0.99
Ankle	A–P	Start	0.89	0.61~0.97
		Maximum	0.91	0.67~0.98
		End	0.81	0.28~0.95
	M–L	Start	0.98	0.91~0.99
		Maximum	0.81	0.27~0.95
		End	0.81	0.29~0.95
	Vertical	Start	0.83	0.36~0.95
		Maximum	0.88	0.57~0.97
		End	0.85	0.44~0.96

**Table 3:** Concurrent validity of MediaPipe for joint positions of the healthy group.

Joint	Axis	Points	*r*	95% CI	RMSE (mm)	Bias (mm)
Hip	A–P	Start	0.99 **	1.04~1.41	51.20	−49.89
		Maximum	0.99 **	0.67~1.46	48.37	−37.52
		End	0.99 **	1.05~1.39	49.31	−48.64
	M–L	Start	0.89 **	0.06~1.08	25.78	−21.40
		Maximum	0.90 **	0.15~1.25	16.51	−3.93
		End	0.90 **	0.07~1.14	25.34	−21.54
	Vertical	Start	0.98 **	0.83~1.08	14.06	0.30
		Maximum	0.98 **	0.86~1.20	19.28	−14.81
		End	0.98 **	0.81~1.09	14.97	−1.56
Knee	A–P	Start	0.99 **	0.88~1.30	30.12	13.63
		Maximum	0.99 **	0.76~1.24	31.40	21.46
		End	0.99 **	0.81~1.29	30.62	9.56
	M–L	Start	0.99 **	0.42~1.46	11.52	6.34
		Maximum	0.98 **	0.04~1.53	18.24	11.27
		End	0.98 **	0.34~1.44	11.87	5.75
	Vertical	Start	0.97 **	0.85~1.23	21.70	−21.70
		Maximum	0.98 **	0.79~1.05	16.97	8.13
		End	0.96 **	0.77~1.23	22.63	−21.53
Ankle	A–P	Start	0.93 **	0.59~1.09	25.90	−0.31
		Maximum	0.92 **	0.52~1.03	47.30	44.13
		End	0.94 **	0.65~1.13	22.62	−4.16
	M–L	Start	0.93 **	0.60~1.14	8.74	0.74
		Maximum	0.85 **	0.41~1.16	15.39	11.87
		End	0.93 **	0.61~1.14	8.54	1.57
	Vertical	Start	0.97 **	0.71~1.01	18.49	2.61
		Maximum	0.97 **	0.73~1.08	36.47	36.35
		End	0.97 **	0.70~1.01	19.07	2.62

** Statistically significant correlation (*p* < 0.01); *r* = Pearson’s correlation coefficient; A–P = anterior–posterior; M–L = medio–lateral; SD = standard deviation; CI = confidence interval.

**Table 4:** Concurrent validity of MediaPipe for joint positions of the DLD (degenerative lumbar disease) group.

Joint	Axis	Points	*r*	95% CI	RMSE (mm)	Bias (mm)
Hip	A–P	Start	0.93 **	0.74~1.49	49.56	−19.32
		Maximum	0.86 **	0.58~1.72	45.68	−10.49
		End	0.93 **	0.75~1.48	51.39	−22.03
	M–L	Start	0.90 **	0.30~0.69	24.70	−18.55
		Maximum	0.87 **	0.36~0.96	13.62	−3.91
		End	0.93 **	0.33~0.65	23.72	−18.43
	Vertical	Start	0.97 **	1.03~1.54	18.28	3.21
		Maximum	0.99 **	0.88~1.11	19.49	−13.10
		End	0.98 **	1.06~1.50	15.70	0.42
Knee	A–P	Start	0.85 **	0.34~1.07	33.01	−17.80
		Maximum	0.92 **	0.70~1.43	35.27	25.71
		End	0.84 **	0.38~1.25	31.18	−23.35
	M–L	Start	0.86 **	0.35~1.01	9.78	−0.74
		Maximum	0.87 **	0.63~1.68	14.41	10.43
		End	0.93 **	0.55~1.06	7.88	0.49
	Vertical	Start	0.98 **	0.74~1.03	30.55	−28.98
		Maximum	0.99 **	0.89~0.95	15.54	−3.69
		End	0.99 **	0.76~0.98	29.95	−27.34
Ankle	A–P	Start	0.87 **	0.51~1.38	24.80	−16.94
		Maximum	0.90 **	0.49~1.14	54.55	53.47
		End	0.87 **	0.47~1.31	22.79	−9.83
	M–L	Start	0.99 **	0.97~1.27	12.01	4.80
		Maximum	0.93 **	0.79~1.52	23.29	23.29
		End	0.98 **	0.96~1.30	13.67	6.57
	Vertical	Start	0.99 **	0.96~1.05	15.22	15.22
		Maximum	0.99 **	0.85~1.01	41.83	40.56
		End	0.99 **	0.95~1.05	16.54	16.54

** Statistically significant correlation (*p* < 0.01); *r* = Pearson’s correlation coefficient; A–P = anterior–posterior; M–L = medio–lateral; SD = standard deviation; CI = confidence interval.

## Data Availability

The original contributions presented in this study are included in the article. Further inquiries can be directed to the corresponding author.
